# Mechanical Unloading Activates FoxO3 to Trigger Bnip3‐Dependent Cardiomyocyte Atrophy

**DOI:** 10.1161/JAHA.113.000016

**Published:** 2013-04-24

**Authors:** Dian J. Cao, Nan Jiang, Andrew Blagg, Janet L. Johnstone, Raj Gondalia, Misook Oh, Xiang Luo, Kai‐Chun Yang, John M. Shelton, Beverly A. Rothermel, Thomas G. Gillette, Gerald W. Dorn, Joseph A. Hill

**Affiliations:** 1 Department of Internal Medicine (Cardiology), University of Texas Southwestern Medical Center, Dallas, Texas (D.J.C., N.J., A.B., J.L.J., R.G., X.L., K.C.Y., J.M.S., B.A.R., T.G.G., J.A.H.); 2 Department of Molecular Biology, University of Texas Southwestern Medical Center, Dallas, Texas (B.A.R., J.A.H.); 3 Department of Biochemistry and Molecular Biology, Indiana University, Indianapolis, IN (M.O.); 4 Center for Pharmacogenomics, Department of Internal Medicine, Washington University School of Medicine, St. Louis, MO (G.W.D.)

**Keywords:** autophagy, cardiac atrophy, cardiac hypertrophy, FoxO3, heart failure

## Abstract

**Background:**

Mechanical assist device therapy has emerged recently as an important and rapidly expanding therapy in advanced heart failure, triggering in some patients a beneficial reverse remodeling response. However, mechanisms underlying this benefit are unclear.

**Methods and Results:**

In a model of mechanical unloading of the left ventricle, we observed progressive myocyte atrophy, autophagy, and robust activation of the transcription factor FoxO3, an established regulator of catabolic processes in other cell types. Evidence for FoxO3 activation was similarly detected in unloaded failing human myocardium. To determine the role of FoxO3 activation in cardiac muscle in vivo, we engineered transgenic mice harboring a cardiomyocyte‐specific constitutively active FoxO3 mutant (*caFoxO3*^*flox*^;α*MHC‐Mer‐Cre‐Mer*). Expression of caFoxO3 triggered dramatic and progressive loss of cardiac mass, robust increases in cardiomyocyte autophagy, declines in mitochondrial biomass and function, and early mortality. Whereas increases in cardiomyocyte apoptosis were not apparent, we detected robust increases in Bnip3 (Bcl2/adenovirus E1B 19‐kDa interacting protein 3), an established downstream target of FoxO3. To test the role of Bnip3, we crossed the *caFoxO3*^*flox*^;α*MHC‐Mer‐Cre‐Mer* mice with Bnip3‐null animals. Remarkably, the atrophy and autophagy phenotypes were significantly blunted, yet the early mortality triggered by FoxO3 activation persisted. Rather, declines in cardiac performance were attenuated by proteasome inhibitors. Consistent with involvement of FoxO3‐driven activation of the ubiquitin‐proteasome system, we detected time‐dependent activation of the atrogenes program and sarcomere protein breakdown.

**Conclusions:**

In aggregate, these data point to FoxO3, a protein activated by mechanical unloading, as a master regulator that governs both the autophagy‐lysosomal and ubiquitin‐proteasomal pathways to orchestrate cardiac muscle atrophy.

## Introduction

A striking feature of the heart is its ability to remodel in response to changes in environmental demand. Indeed, the heart is a remarkably plastic organ, capable of growing or shrinking in response to a variety of stimuli.^1^ In recent years, significant strides have occurred in deciphering the progrowth mechanisms of cardiac hypertrophy, yet little is known regarding mechanisms that govern cardiomyocyte atrophy. Unveiling those mechanisms has relevance to a number of clinically important conditions, including bed rest, mechanical support therapy, cancer, glucocorticoid use, and prolonged weightlessness. Further, activation of antigrowth pathways in the hypertrophied heart may be a means to renormalize cardiac mass, improve ventricular relaxation, and diminish excessive filling pressures, which together contribute to the widespread syndrome of heart failure with preserved ejection fraction. Support for this concept derives from clinical studies in which regression of the cardiac mass is associated with reduction of adverse cardiac events.^2–3^

Mechanical unloading of the failing ventricle with a ventricular assist device (VAD) has emerged in recent years as a major therapeutic option. Atrophy of the diseased and typically hypertrophied, left ventricle (LV) is a hallmark feature of VAD therapy. In rare instances, substantial reverse remodeling and recovery of ventricular performance are seen. However, underlying mechanisms are unknown. Regardless, regression of pathological hypertrophy is consistently seen, and activation of catabolic processes is likely to contribute.

Here, we set out to decipher mechanisms of cardiomyocyte atrophy elicited by mechanical unloading of the LV. As part of this, we have focused on FoxO (Forkhead box‐containing O family) transcription factors, proteins implicated in the control of numerous cellular processes, including cell cycle regulation, differentiation, metabolism, proliferation, and survival.^4–6^ Specifically, we dissected the contribution of catabolic events elicited by mechanical unloading and the upstream, transcriptional circuitry that governs their expression.

## Materials and Methods

### Heterotopic Cardiac Transplantation

Surgical transplantation of the heart was accomplished as described previously.^7^ Briefly, the ascending aorta and pulmonary artery of a donor mouse heart were sutured to the inferior abdominal aorta and inferior vena cava, respectively, of a same‐strain recipient. In this way, the recipient animal's native heart perfuses the donor ascending aorta in a retrograde fashion, maintaining the aortic valve closed and directing arterial blood into the coronary circulation. The donor LV is therefore mechanically unloaded but well perfused. After perfusing the donor heart, venous blood exiting the coronary sinus is ejected from the right ventricle into the vena cava to be recirculated back into the recipient circulation. The successful rate of these surgeries is about 90% in our laboratory. Additional detailed Materials and Methods are available at http://jaha.ahajournals.org/content/2/2/e000016.full.

### Statistical Methods

Averaged data (expressed as mean±SD) were analyzed with the unpaired Student *t* test for 2 independent groups, paired *t* test for dependent data, and 1‐way ANOVA followed by post‐hoc tests, such as Bonferroni. For statistical comparisons, a *P* value <0.05 was considered statistically significant. Normality tests were conducted via the Shapiro–Wilk and Anderson–Darling statistics. Assessment of skewness and kurtosis and a quantitative inspection of the closeness of the mean and the median were also used to establish normality. As normality was confirmed (Tables S1), results are presented from parametric statistics. However, all results were confirmed with nonparametric tests. All statistical analyses were performed using Sigma Stat (version 3.1) software.

## Results

### Cardiac Myocyte Autophagy and FoxO3 Are Activated by Mechanical Unloading

**Figure 1.**
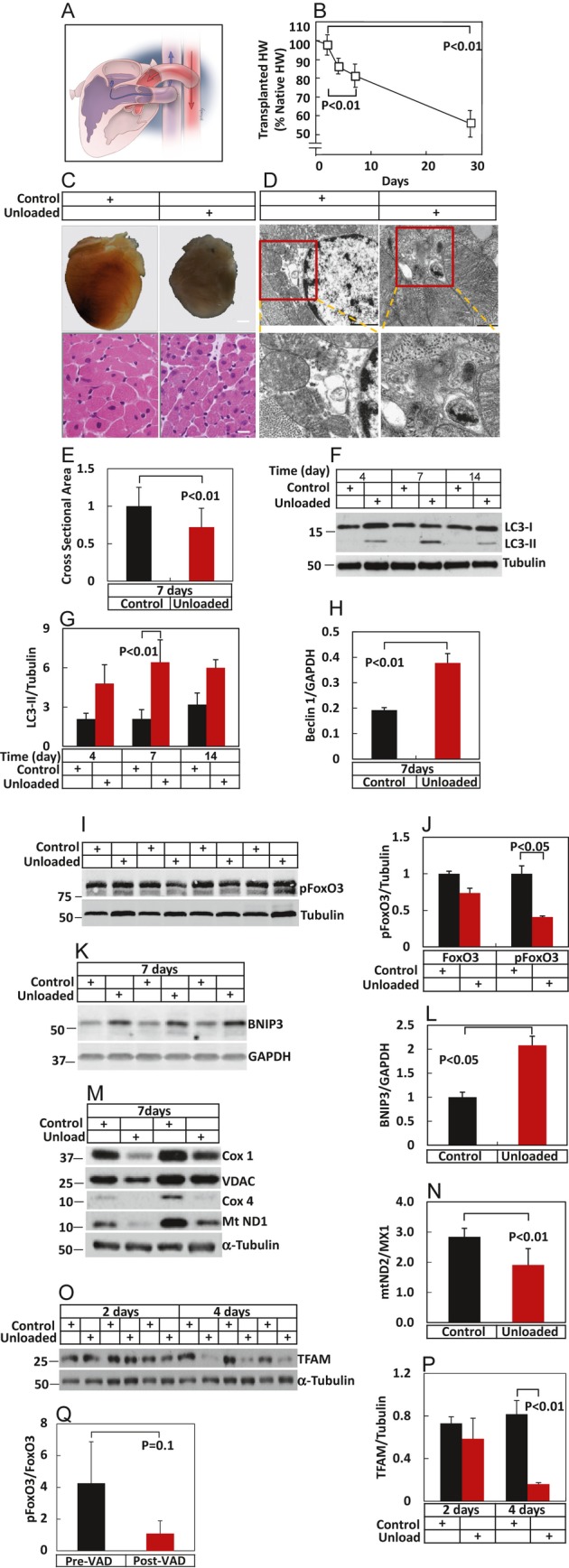
Mechanical unloading of the LV triggers FoxO3 activation and autophagy. A, Schematic diagram of heterotopic cardiac transplantation. B, Time course of cardiac atrophy after transplantation (n=3). C, Whole heart images of native and donor hearts, as well as hematoxylin and eosin–stained LV tissue sections, are shown. D, Increased autophagosome abundance in unloaded LV. E, Cardiomyocyte cross‐sectional areas of representative control and unloaded heart pairs following 7 days of unloading, n=200 myocytes per sample from 2 hearts in each group. F and G, Increased LC3‐II levels were observed in donor hearts after unloading. H, Beclin 1 protein levels were increased in unloaded LV (n=2). I and J, Phosphorylated FoxO3 and total FoxO3 from native and donor hearts after 4 days of unloading. K and L, Bnip3 levels from native and donor hearts after 4 days of unloading. n=3 to 6. M, Mitochondrial protein levels, including Cox1, VDAC, Cox4, and Mt ND1 in control and unloaded hearts 7 days after unloading. Experiments repeated 3 times with similar results. N, Mitochondrial DNA (mtDN2) copy number normalized to nuclear DNA (MX1) in control and unloaded hearts. O, TFAM protein levels in control and unloaded hearts at 2 and 4 days after unloading (n=3 for each group). P, Quantitative data from O. Q, Phosphorylated FoxO3 levels in failing human LV before and after VAD support, n=3. LV indicates left ventricle; HW, heart weight; Cox, cyclooxygenase; VDAC, voltage‐dependent anion channel; mt ND1, mitochondrial NADH dehydrogenase 1; MX1, myxovirus resistance 1; TFAM, transcription factor A, mitochondrial; VAD, ventricular assist device; GAPDH, glyceraldehyde 3‐phosphate dehydrogenase; HE, hematoxylin and eosin; GFP, green fluorescent protein; Myc, myelocytomatosis oncogene; MOI, multiplicity of infection; NC siRNA, negative control siRNA; NS, not significant; MuRF, muscle ring finger.

Atrophy of cardiac muscle is observed in the setting of mechanical unloading, including that elicited by bed rest, prolonged weightlessness, or VAD therapy. In the case of the latter, the failing heart undergoes significant structural and functional changes, including declines in ventricular mass.^8^ To begin to tease out mechanisms contributing to these clinically important changes, we used a model of heterotopic cardiac transplantation. In this model, the heart of 1 mouse (donor) is surgically anastomosed to the abdominal aorta and vena cava of another mouse (native) (Figure 1A). Donor hearts subjected to mechanical unloading did not manifest evidence of injury, and circulating levels of cardiac biomarkers (creatine kinase MB, troponin T) were not detected. Donor hearts, however, manifested a time‐dependent decrease in heart mass (Figure 1B and 1C). On microscopic analysis, myocyte cross‐sectional areas were decreased by 28% (*P*<0.01) at day 7 (Figure 1E). Terminal deoxynucleotidyl transferase–mediated dUTP nick end labeling assay (TUNEL) assays were negative, ruling out a significant increase in apoptosis or other cell injury process associated with DNA fragmentation.

Growth or atrophy of the heart is a consequence of an orchestrated balance between anabolic and catabolic events.^1^ Indeed, the heart's extraordinary capacity for plasticity derives from robust progrowth and antigrowth capabilities, each of which can be activated rapidly. Consistent with a significant contribution of autophagy, an evolutionarily conserved mechanism of regulated cellular cannibalization, we detected an abundance of autophagosomes on electron microscopic examination in unloaded LV (Figure 1D). Increased levels of LC3‐II were detected in protein lysates harvested from LV subjected to 4, 7, and 14 days of unloading, indicative of increased autophagosome formation in atrophying hearts (Figure 1F and 1G). Also indicative of activated autophagic flux, levels of Beclin 1, a protein involved in both early and late autophagosome processing events,^9^ were elevated (Figure 1H).

Studies of skeletal muscle atrophy have uncovered a central role of FoxO factors as master regulators of atrophy‐induced catabolic pathways.^10^ To test for a role of FoxO in unloading‐induced cardiac atrophy, we probed for the phosphorylated, transcriptionally inactive isoform of FoxO3. Here, we uncovered evidence for increased FoxO3 activity as assessed indirectly by decreased levels of FoxO3 protein phosphorylated at Thr32 (Figure 1I and 1J). Consistent with FoxO3 activation, levels of Bnip3 (Bcl2/adenovirus E1B 19‐kDa interacting protein 3), an established FoxO3 downstream target, were also increased by mechanical unloading (Figure 1K and 1L).

As autophagy is a major mechanism underlying the elimination of mitochondria, we evaluated levels of 4 mitochondrial proteins, Cox 1 (cyclooxygenase 1), VDAC (voltage‐dependent anion channel), Cox4 (cyclooxygenase 4), and ND1 (NADH dehydrogenase 1). In each case, we noted significant declines in steady‐state protein levels, consistent with activation of autophagy (Figure 1M). Levels of the mitochondrial protein TFAM (transcription factor A, mitochondrial) were significantly diminished by day 4 of unloading (Figure 1O and 1P). Further, quantification of DNA abundance of a mitochondrial gene (*mtND2* [mitochondrial NADH dehydrogenase 1]) relative to a nuclear gene (*MX1* [myxovirus resistance 1]) revealed additional evidence of significant declines in mitochondrial biomass (Figure 1N). In aggregate, these data suggest that FoxO3‐dependent catabolic events participate in the reverse remodeling that occurs in the mechanically unloaded LV.

To test for FoxO3‐dependent events in human myocardium, we evaluated paired samples of failing human LV harvested at the time of VAD implantation and at the time of VAD removal. In the samples (3 pre‐VAD, 3 post‐VAD), we observed a trend toward declines in pFoxO3 levels, suggestive of FoxO3 activation in the mechanically unloaded human heart (Figure 1Q).

### Cardiomyocyte‐Restricted *caFoxO3* Transgenic Mice

These data suggest that unloading‐induced activation of FoxO3 participates in the ventricular remodeling response. To test this, we engineered a line of compound transgenic mice that express a constitutively active mutant of FoxO3 (caFoxO3) in cardiac myocytes (Figure S1A). Myc‐tagged FoxO3 was rendered constitutively active by mutations at 3 Akt phosphorylation sites,^11^ and a 5′ Stop codon was flanked with *loxP* sites. Cardiac‐specific activation of the *FoxO3* transgene was achieved by crossing these mice with a line of mice expressing Cre recombinase flanked by mutated estrogen receptor ligand–binding domains (*MerCreMer* [*MCM*]) driven by the α*MHC* promoter.^12^ Exposure of these double transgenic *caFoxO3*^*flox*^*;αMHC‐MCM* mice (hereafter termed *caFoxO3;MCM*) to tamoxifen (20 mg/kg IP×3 days) was sufficient to excise the Stop codon and trigger caFoxO3 expression (Figure S1B).

*caFoxO3;MCM* mice manifested a normal life span and were indistinguishable from animals expressing either transgene alone and from wild‐type (WT) mice. One additional caFoxO3 transgenic line was identified, analyzed, and yielded similar results. To test for transcriptional activity of the transgene following exposure to tamoxifen, we evaluated multiple downstream targets, finding that protein levels of p27 and Bnip3 were elevated (Figure S1B and S1C).

Akt is subject to regulation by FoxO transcription factors.^13–14^ Indeed, we reported previously that FoxO factors are capable of targeting calcineurin,^15^ which, in turn, dephosphorylates Akt.^16^ Consistent with this, we found increased levels of phosphorylated Akt in *caFoxO3;MCM* hearts (Figure S1B and S1C).

To determine the effects of caFoxO3 overexpression on levels of endogenous FoxO3, we measured both proteins by immunoblot analysis of lysates harvested from hearts of *caFoxO3;MCM* mice and their control littermates. Compound transgenic animals exposed to tamoxifen manifested an 8‐fold increase in total FoxO3 protein (Figure S1D and S1E). Transgene expression had little effect on endogenous FoxO3 levels. To determine the efficiency of Cre‐mediated recombination (and, hence, caFoxO3 expression), we isolated adult cardiac myocytes from *caFoxO3;MCM* mice and their control littermates after 2‐day treatment with tamoxifen. Immunostaining for myc‐tagged caFoxO3 demonstrated that 80% to 90% of cardiomyocytes expressed the transgene, as evidenced by positive nuclear staining (Figure S1F).

### *caFoxO3* Provokes Robust Cardiac Atrophy

**Figure 2.**
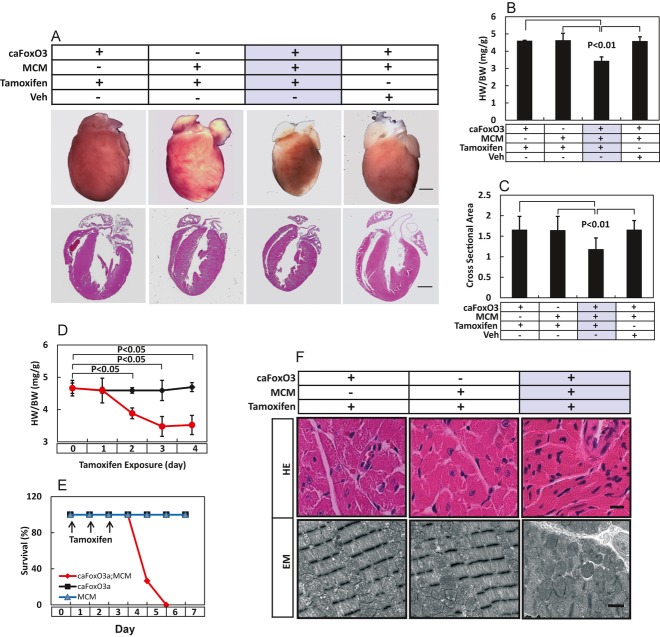
Cardiac atrophy induced by FoxO3. A, Whole hearts and 4‐chamber sections of hearts from *caFoxO3:MCM* mice and control littermates (harboring either caFoxO3 or MCM but not both) after 3 tamoxifen injections. A heart from a *caFoxo3;MCM* mouse treated with vehicle is also depicted. B, Heart weight–to–body weight ratios in each groups of animals (n=5 to 11). C, Cardiomyocyte cross‐sectional areas from these groups of hearts (n=60 to 65). D, Time course of declines in cardiac mass in *caFoxO3;MCM* mice measured after 0, 3, 24, 48, and 72 hours of tamoxifen exposure (n=3 to 5). E, Survival curves of *caFoxO3;MCM* mice and their control littermates after tamoxifen injection (n=8 to 15). The control groups overlapped throughout the experiment, as their survival was 100%. F, Hematoxylin and eosin staining of LV sections and EM‐based ultrastructure from corresponding groups of mice (n=3). MCM indicates *MerCreMer*; HW, heart weight; BW, body weight; LV, left ventricle; EM, electron microscopic.

To evaluate the effects of FoxO3 expression in the heart, cohorts of mice harboring *caFoxO3*,* MCM*, or both (*caFoxo3;MCM*) were treated with tamoxifen (20 mg/kg×3 days). Compound transgenic *caFoxo3;MCM* mice were subjected to vehicle treatment, as well as an additional control. On day 4, the animals were killed, and hearts were subjected to necropsy analysis. Animals expressing caFoxO3 manifested dramatic cardiac atrophy, which was absent from all other lines (Figure 2A). Within 3 days of transgene activation, heart weight normalized to body weight (HW/BW) in *caFoxo3;MCM* mice (3.3±0.35 mg/g, n=11, *P*<0.01) was decreased 30% (±2, n=11, *P*<0.01) relative to compound transgenics exposed to vehicle (4.66±0.24 mg/g, n=4) (Figure 2B). Myocyte cross‐sectional areas (measured with ImageJ software [National Institutes of Health] and expressed with arbitrary units) were diminished to a similar degree (30±3%, n=70, *P*<0.01), suggesting that myocyte atrophy, as opposed to cell dropout, was the predominant mechanism underlying decreased cardiac mass (Figure 2C).

In a second series of experiments, time‐course analyses were performed to determine the natural history of myocyte‐restricted FoxO3 overexpression. These studies revealed robust and statistically significant declines in HW/BW as early as 24 hours after the first injection of tamoxifen (Figure 2D). Coincident with this progressive atrophy phenotype, early lethality was observed. Remarkably, survival of *caFoxo3;MCM* was 100% on day 4 but declined precipitously thereafter, such that survival on day 6 was 0% (Figure 2E). Cardiac function measured by M‐mode echocardiography progressively declined in *caFoxO;MCM* mice (Figure S2). Histological analyses of myocardial tissues from hearts on day 4 revealed diminished myocyte size in *caFoxO3;MCM* mice and pyknotic nuclei (Figure 2F). Myocardial ultrastructure revealed disorganized mitochondria and sarcomeres, suggestive of activated catabolic pathways (Figure 2F).

### FoxO3‐Driven Activation of Cardiomyocyte Autophagy In Vivo

**Figure 3.**
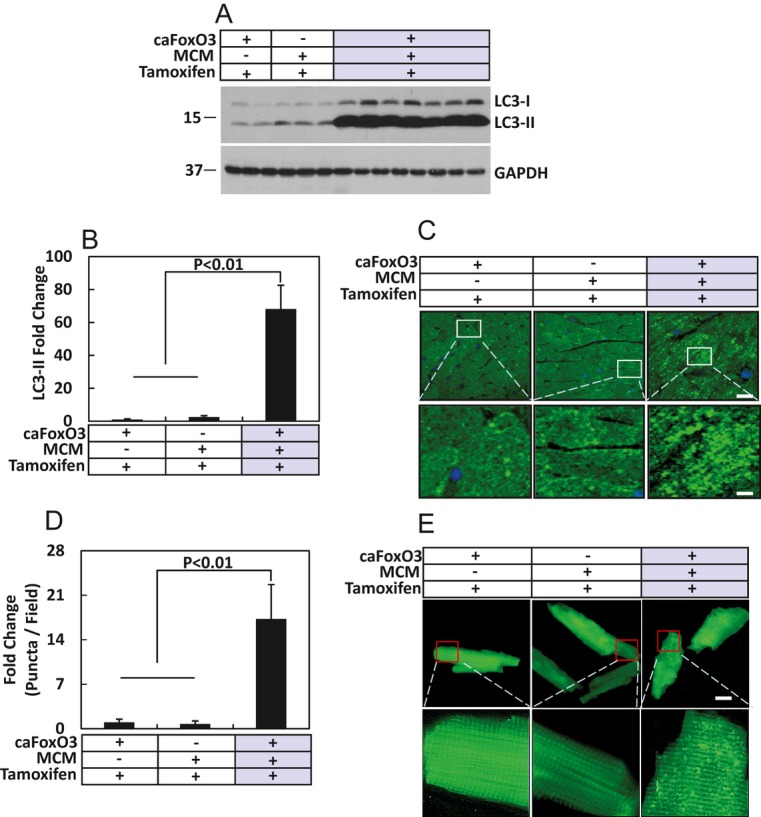
Activation of autophagy in FoxO3‐expressing myocardium. A, The autophagy marker LC3‐II was quantified in total protein lysates extracted from hearts of the mouse cohorts indicated (n=3 to 7). B, Mean data from experiments depicted in A. C, Confocal images obtained from immunohistochemical studies performed on α*MHC‐GFP‐LC3*;*caFoxO3;MCM* using an anti–green fluorescent protein (GFP) antibody. Positive staining as puncta indicates autophagosome‐localized GFP‐LC3 (n=3). D, Quantification of GFP‐LC3 puncta from experiments as depicted in C. E, Single cardiac myocytes were isolated from animals as indicated 48 hours postexposure to tamoxifen. GFP fluorescence of the GFP‐LC3 puncta (without staining with anti‐GFP antibody) was visualized under confocal microscopy (n=3). MCM indicates *MerCreMer*.

FoxO transcription factors govern a wide range of processes, including catabolic events in skeletal muscle^10^ and neonatal cardiomyocytes.^17^ To determine whether FoxO3 activation confers similar effects in adult cardiac muscle in vivo, we tested for evidence of activation of autophagic flux mechanisms. First, levels of LC3‐II, a marker indicative of autophagosome abundance,^18^ were evaluated by Western blot of proteins harvested from *caFoxO3;MCM* mice and littermate controls (each exposed to tamoxifen) (Figure 3A and 3B). Mean data indicated that LC3‐II levels were increased 60‐fold (±4, n=7, *P*<0.01) in *caFoxO3;MCM* mice relative to littermate controls.

To evaluate further for evidence of autophagic activation, we crossed the compound transgenic *caFoxO3;MCM* mice with mice harboring a green fluorescent protein (GFP)‐tagged LC3 transgene; these latter “autophagy reporter mice” provide a reliable means of quantifying autophagy levels and determining the cellular origin of the LC3 signal.^19^ Hearts from animals in which the *caFoxO3* transgene had been activated for 3 days were evaluated by immunohistochemistry with an anti‐GFP antibody and confocal microscopy. We detected a dramatic increase in GFP‐LC3 puncta in *caFoxO3;MCM* mice compared with littermates lacking either the caFoxO3 transgene or the MCM transgene (Figure 3C). And as the GFP‐LC3 construct is expressed under the control of the cardiac myocyte–specific αMHC promoter, these data confirm that the signal—indicative of autophagosome accumulation—derives from cardiac myocytes. Quantification of GFP‐LC3 puncta revealed 17‐fold (±1.5, *P*<0.01, n=3) higher levels of GFP‐LC3–tagged autophagosomes in hearts expressing the *caFoxO3* protein compared with hearts from single transgene lines (Figure 3D). Similar findings were obtained in cardiac myocytes isolated from mice from each of these lines after 2 days of tamoxifen exposure (Figure 3E).

Detection of double‐membrane or multimembrane vesicles is the sine qua non of autophagosome accumulation. In hearts harboring either the MCM transgene alone or the caFoxO3 transgene alone (and exposed to tamoxifen), we detected double‐membrane autophagosomes in a predominantly perinuclear distribution (Figure S3A) consistent with the expected levels of basal autophagic flux. The appearance and abundance of these vesicles were similar to those seen in tamoxifen‐treated WT mice. In contrast, a vast abundance of autophagosomes was readily detected in *caFoxO3;MCM* mice in which the *caFoxO3* transgene had been activated for 3 days (Figure S3B through S3E). In these hearts, autophagosomes were detected in the process of engulfing a mitochondrion (Figure S3B), after a mitochondrion had been completely surrounded (Figure S3C), or at later stages where vesicular contents had been degraded (Figure S3D and S3E).

### Evidence of Mitochondrial Mass Reduction

Turnover of mitochondria occurs via autophagic degradation.^20^ To test further for activation of autophagic mechanisms and effects on mitochondrial biomass, we used dyes specific for mitochondrial abundance (MitoTraker Green [MTG] and Nonyl acridine orange [NAO]) or for functionally intact mitochondria as evidenced by preservation of mitochondrial membrane potential (tetramethyl rhodamine methyl ester [TMRM]). First, we isolated adult cardiac myocytes from *caFoxO3;MCM* mice after they had been exposed to tamoxifen for 2 days. In these hearts, we detected evidence for significant decreases in both mitochondrial mass (Figure S3F and S3G) and intact mitochondria (Figure S3H). Exposure to *p*‐trifluoromethoxy carbonyl cyanide phenylhydrazone (FCCP), a mitochondrial protonophore that causes mitochondrial depolarization, abolished TMRM accumulation in cardiac myocytes (Figure S3H). Consistent findings were obtained when we evaluated mitochondrial DNA (mtDNA) in relation to nuclear DNA. Quantitative real‐time (RT)–polymerase chain reaction (PCR) with 2 independent mitochondrial gene‐specific primers (NADH2, NADH1) relative to 2 independent nuclear DNA primers (MX1, H19) revealed statistically significant declines in mitochondrial biomass in hearts expressing the *caFoxO3* transgene (Figure S3I).

FoxO factors are transcriptional coactivators involved in the expression of numerous genes, including those regulating autophagy.^21^ As the autophagic machinery is governed by 32 autophagy‐related (*atg*) genes,^22^ we set out to test for possible effects of FoxO3 on *atg* gene expression. Time course analysis using quantitative RT‐PCR performed on RNA harvested from heart tissue revealed that *Atg8* (gene coding for LC3) was upregulated significantly as early as 24 hours after transgene activation (Figure S3J). As expected, no changes in *atg* gene expression were seen in tamoxifen‐treated *MCM* mice (Figure S3K).

Previous studies reported that caFoxO3 increased autophagic flux.^23^ We thus investigated the impact of FoxO3 on autophagic flux using an RNA interference knockdown strategy. Two sequence‐independent silent interfering RNAs (siRNAs) targeting FoxO3 were engineered. Knocking down endogenous FoxO3 in cardiomyocytes significantly decreased LC3‐II levels at baseline as well as in cells treated with bafilomycin (Figure S4A through S4C). Together, these data, coupled with our findings of increased Beclin 1 expression (Figure 1H) and prior reports in the literature,^23^ indicate that FoxO3 activates cardiomyocyte autophagic flux.

### Absence of Apoptotic Cell Death in *caFoxO3* Hearts

The presence of pyknotic nuclei in caFoxO3‐expressing hearts suggested that mechanisms other than autophagy are active. To test for the possible involvement of apoptosis, we examined the tissue sections for evidence of apoptotic morphology, such as nuclear fragmentation. This was not detected in either hematoxylin and eosin–stained sections (Figure 2F) or on electron microscopic studies (Figure S3C). Immunoblotting for caspases 3, 6, 9, and 12 failed to reveal caspase cleavage, a marker of apoptosis (Figure S5A and S5B), whereas positive controls revealed the cleaved isoforms. Consistent with this, TUNEL assays on myocardial tissue sections obtained from *caFoxO3;MCM* mice and their control littermates revealed only rare TUNEL‐positive myocytes in all groups (Figure S5C), although they were readily detected in positive controls. In fact, TUNEL‐positive cells were so rare, and similar across the 3 genotypes, that rigorous statistical comparisons were difficult to perform. Together, these data suggest strongly that loss of cardiac mass in caFoxO3‐expressing transgenics is not due to apoptotic cell death.

### Bnip3 Is Required for FoxO3‐Driven Cardiomyocyte Autophagy

**Figure 4.**
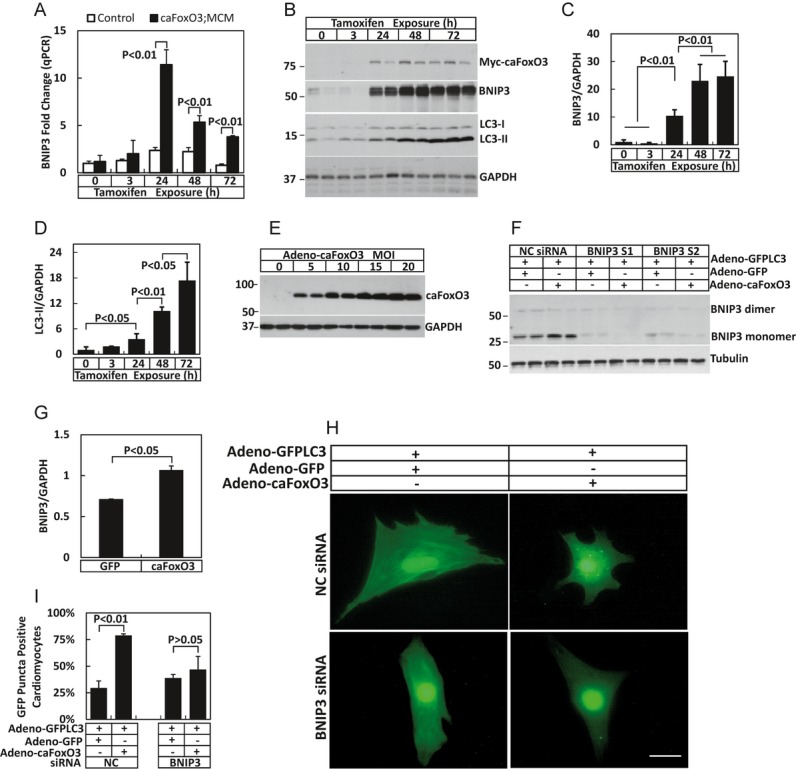
Activation of autophagy closely correlated with FoxO3‐elicited Bnip3 up‐regulation. A, Bnip3 transcript levels estimated by quantitative RT‐PCR at different time points after tamoxifen injection (n=3 to 5). B, Time course of Bnip3 and LC3‐II protein levels after inducing caFoxO3 expression (n=3 to 5). Mean data depicting Bnip3 (C) and LC3‐II protein levels (D). E, caFoxO3 protein levels in neonatal rat ventricular myocytes after infection with adenoviral green fluorescent protein (GFP)‐caFoxO3 as a function of increasing MOI (n=3). F, Efficacy of protein knockdown in neonatal rat ventricular myocytes incubated with scrambled siRNA (NC siRNA) or siRNA targeting Bnip3. G, Bnip3 protein levels in GFP or caFoxO3 adenovirus‐infected NRVMs. H, NRVM co‐infected with adeno‐GFP‐LC3 plus adeno‐caFoxO3 or adeno‐GFP. GFP‐LC3 puncta in these NRVMs were visualized by immunofluorescence (n=4). I, Percentage of GFP‐LC3 puncta‐positive NRVMs (n=4). Bnip3 protein levels after siRNA knockdown with 2 sequence‐independent RNAi constructs (Bnip3 S1, Bnip3 S2) (n=3). qRT‐PCR indicates quantitative real‐time–polymerase chain reaction; MCM,* MerCreMer*; NRVMs, neonatal rat ventricular myocytes.

In our model of mechanical unloading, we observed robust increases in Bnip3 (Figure 1K), a proapoptotic BH3‐only protein capable of inducing autophagy, mitochondrial dysfunction, and turnover in the context of several diseases, including cancer and cardiovascular disease.^24–25^ Similarly, Bnip3 was dramatically upregulated in *FoxO3*‐expressing hearts (Figure S1B), so we set out to evaluate its possible role as a required, downstream effector of FoxO3‐dependent cardiomyocyte autophagy. First, we characterized the kinetics of Bnip3 expression as a function of FoxO3 activation. Interestingly, Bnip3 transcript levels peaked at 24 hours, following only a single dose of tamoxifen (Figure 4A). Bnip3 protein levels were similarly elevated at 24 hours (Figure 4B and 4C) in close temporal relation with LC3‐II (Figure 4B and 4D).

To evaluate the functional role of FoxO3‐driven Bnip3 expression, we used 2 independent siRNA constructs targeting Bnip3 in neonatal rat ventricular myocytes (NRVMs) in culture and examined autophagic activity induced by overexpressed caFoxO3. First, we confirmed caFoxO3 expression (Figure 4E) and evaluated each siRNA reagent for Bnip3 knockdown (Figure 4F). Then, NRVMs were infected with a caFoxO3‐expressing adenovirus after Bnip3 had been depleted with either of the 2 sequence‐independent siRNAs. By coinfecting the cells with a GFP‐*LC3* virus, we quantified the autophagic activity as GFP‐LC3–positive puncta (indicative of autophagosomes). As expected, FoxO3 expression in NRVMs elicited significant increases in Bnip3 protein levels (Figure 4G). Consistent with our in vivo results, overexpression of FoxO3 increased autophagy in NRVMs, and this induction of autophagy was significantly suppressed by Bnip3 knockdown (Figure 4H and 4I). Together, these data are consistent with a model in which Bnip3 is a required downstream effector of FoxO‐driven autophagic flux.

### FoxO3‐Driven Bnip3 Is Required for Autophagic Atrophy

**Figure 5.**
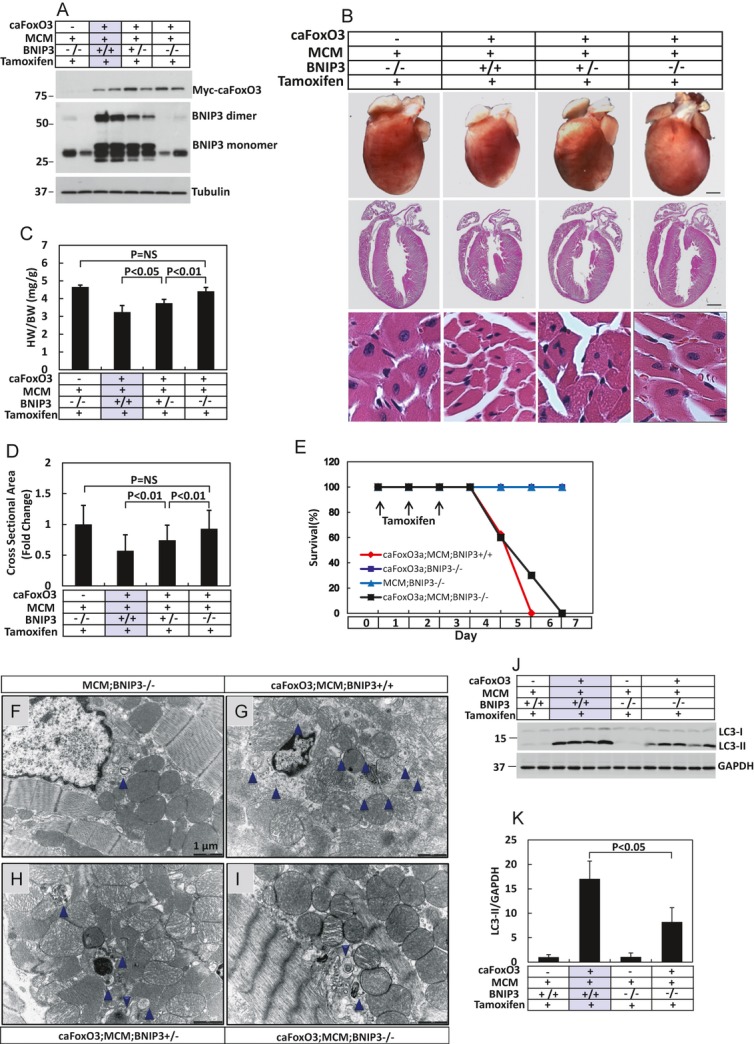
Silencing of Bnip3 gene rescued the cardiac atrophy phenotype induced by caFoxO3. A, Bnip3 protein levels in mouse cohorts of *Bnip3 WT*,* Bnip3±,* or *Bnip3*^*−/−*^ lines (n=2). B, Representative whole heart images, their corresponding 4‐chamber sections, and hematoxylin and eosin staining from animal cohorts as indicated. C, Gravimetric analysis of HW/BW in these mice (n=5 to 9). D, Myocyte cross‐sectional areas from mouse groups as indicated (n=70 to 79). E, Survival curve of animal cohorts with the genetic manipulations indicated. The 2 control groups (caFoxO3;BNIP3^−/−^ and MCM;BNIP3^−/−^) overlapped throughout the experiment, as their survival was 100%. F through I, EM images depicting autophagosome abundance in these hearts. J, Immunoblots of LC3‐II levels from mouse cohorts as indicated (n=2 to 5). K, Mean data of the LC3‐II levels. HW indicates heart weight; BW, body weight; MCM,* MerCreMer*; EM, electron microscopic.

To evaluate the role of FoxO3‐driven Bnip3 expression in the cardiac atrophy phenotype of FoxO3‐activated mice, we crossed *caFoxO3;MCM* mice with *Bnip3*^*−/−*^ mice^26^ to generate *caFoxO3;MCM;Bnip3*^*−/−*^ animals. Identical to the protocol used earlier, the *FoxO3* transgene was activated (3 tamoxifen injections), and hearts were harvested on day 4. As expected, FoxO3‐driven increases in Bnip3 protein were absent in Bnip3‐mutant hearts (Figure 5A). Remarkably, we observed dramatic rescue of the atrophy phenotype in FoxO3‐activated hearts depleted of Bnip3 (Figure 5B and 5C). HW/BW ratios revealed a statistically robust, dose‐responsive relationship between Bnip3 alleles and atrophy rescue (Figure 5C). Measurements of myocyte cross‐sectional areas revealed similar findings: Bnip3 allele–dependent rescue of FoxO3‐driven cardiomyocyte atrophy (Figure 5D). Tamoxifen did not alter survival, HW/BW ratios, or myocyte cross‐sectional areas in *Bnip3*^*−*/*−*^ mice harboring the *MCM* transgene alone (Figure 5E). However, remarkably, rescue of the cardiac atrophy response did not alter the early mortality seen in *caFoxO3;MCM;Bnip3*^*−/−*^ mice (Figure 5E). Ultrastructural analyses confirmed marked reduction in autophagosome abundance in hearts deficient in Bnip3 (Figure 5F through 5I). FoxO3‐dependent increases in LC3‐II were blunted >50% in *Bnip3*^*−/−*^ hearts (Figure 5J and 5K). These findings suggest strongly that FoxO‐elicited cardiomyocyte atrophy and autophagy require Bnip3 activation, yet the early mortality phenotype involves mechanisms independent of Bnip3, autophagy, and myocardial atrophy.

### FoxO3‐Dependent Activation of the Ubiquitin‐Proteasome System

**Figure 6.**
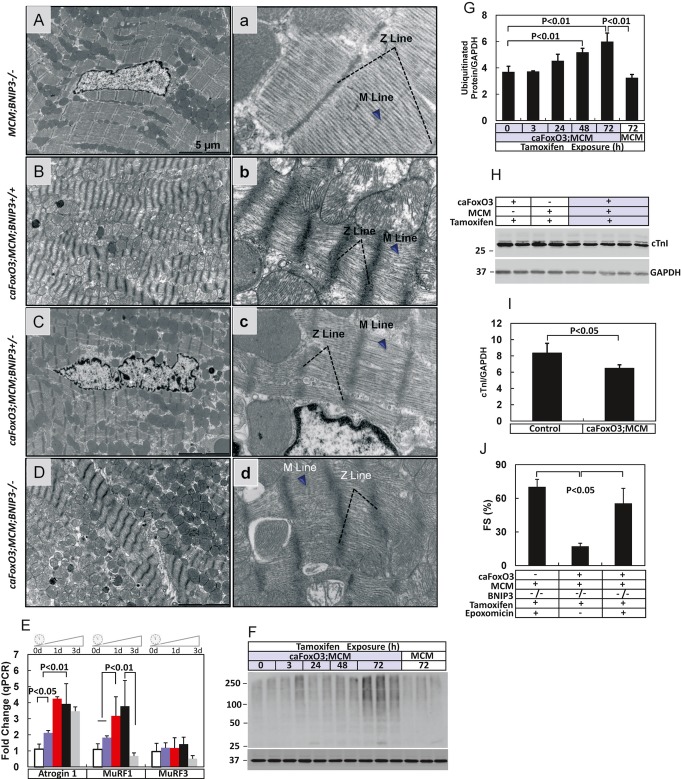
Activation of protein ubiquitination pathways and sarcomere derangement by FoxO3. A through D, Sarcomere ultrastructure in mouse cohorts as indicated (n=2 to 3). a through d, High‐magnification images of micrographs depicted in panels A through D. E, RNA levels of E3 ubiquitin ligases *Atrogin1, Murf1,* and *Murf3* measured by quantitative RT‐PCR at different time points (0, 3, 24, 48, and 72 hours) after tamoxifen treatment (n=3 to 5). F, Accumulation of ubiquitinated proteins in the hearts of *caFoxO3;MCM* mice following exposure to tamoxifen (n=3). G, Mean data from experiments as depicted in F. H, Cardiac troponin I (cTnI) levels in *caFoxO3;MCM* mice and control littermates (n=2 to 5). I, Mean cTnI levels from mouse cohorts as indicated. J, Ventricular function measured as percent fractional shortening by echocardiography in mice treated with or without epoxomicin at 0.5 mg/kg per day for 3 days (n=3). MCM indicates *MerCreMer*; qRT‐PCR, quantitative real‐time–polymerase chain reaction; cTnI, cardiac troponin I; FS, fractional shortening.

To test for mechanisms other than autophagy‐driven atrophy that contribute to the mortality phenotype, we reevaluated cardiac myocyte ultrastructure in *caFoxO3;MCM;Bnip3*^*+/+*^, *caFoxO3;MCM;Bnip3*^*+/−*^, *caFoxO3;MCM;Bnip3*^*−/−*^, and *MCM;Bnip3*^*+/+*^ hearts. These studies revealed evidence of sarcomeric disruption in FoxO3‐expressing myocytes, regardless of the Bnip3 genotype; shortened sarcomere length, disappearance of the M line, a diffuse Z line, and disorganized myofibrillar proteins were readily detected (Figure 6A through 6D). As these sarcomeric elements are targets of the ubiquitin‐proteasome system (UPS) cascade of protein catabolism, and as FoxO3 is an established activator of the UPS,^10^ we set out to test for involvement of UPS mechanisms.

Ubiquitin ligase–dependent protein ubiquitylation is an early step in UPS‐dependent protein catabolism. Consistent with activation of the UPS, we found that FoxO3 elicited rapid increases in the abundances of *Atrogin1* and *Murf1*, 2 major ubiquitin ligases known to target the sarcomere (Figure 6E). Consistent with specificity for FoxO3‐dependent activation, *Murf3,* an E3 ligase that has not been established as a FoxO3 downstream target,^27^ was not increased (Figure 6E). Cellular levels of ubiquitylated proteins were increased in FoxO3‐expressing myocardium (Figure 6F and 6G). Finally, as cardiac troponin I (cTnI) is degraded by the UPS in a MuRF1‐dependent manner,^28^ we evaluated the levels of this protein, noting significant FoxO3‐dependent declines (Figure 6H and 6I). Together, these data point to UPS‐dependent catabolic pathways as downstream of FoxO3 activation that appear to contribute importantly to sarcomere integrity but not the atrophy phenotype.

To determine the role of UPS mechanisms in the FoxO3‐elicited phenotype, we tested whether proteasome inhibition could prevent the dramatic declines in cardiac function and early mortality in these animals. To do this, we treated mice with epoxomicin 0.5 mg/kg (SQ in 10% DMSO) starting at the time of transgene activation and ending simultaneous with the end of tamoxifen treatment (total 3 days). Cardiac function, evaluated by echocardiography 3 days later, was significantly improved in mice treated with epoxomicin (Figure 6J), and mortality was delayed (Figure S6). These data, then, suggest that activation of the proteasomal pathway participates importantly in FoxO‐elicited deterioration in cardiac function.

## Discussion

Atrophic remodeling of the LV occurs in numerous clinically relevant contexts, yet underlying molecular mechanisms are ill defined. In recent years, mechanical support of the failing LV has emerged as a major therapeutic strategy, triggering both beneficial and untoward processes. Here, we present evidence for a critical role of FoxO3, a transcription factor involved in numerous cellular processes, including catabolic events. FoxO3 is activated in a model of left ventricular unloading analogous to that which occurs clinically in patients subjected to VAD therapy, as well as in samples of unloaded human myocardium. Forced expression of FoxO3 in cardiomyocytes was sufficient to recapitulate the autophagic, atrophic, and mitochondria‐depleting phenotypes observed in mechanically unloaded LV. Importantly, each of these phenotypes was rescued in hearts in which the gene coding for Bnip3 was silenced, pointing to a critical role for this Bcl2 family protein. With time, however, FoxO3 overexpression ultimately provoked sarcomere degradation, LV dysfunction, and mortality, phenotypes that were not rescued by Bnip3 inactivation. Rather, these latter deleterious events derived from FoxO3‐dependent activation of the UPS and were antagonized by protease inhibition. Together, these findings point to FoxO3 as a master regulator of unloading‐induced catabolic processes in the myocardium (Figure S7). They go on to suggest that beneficial and deleterious remodeling events can be dissociated for potential therapeutic benefit.

### Myocardial Atrophy

Hemodynamic unloading of the LV, as occurs with bed rest, prolonged weightlessness, or VAD therapy, can lead to declines in cardiac mass of as much as 30%. Remarkably, this myocardial atrophy response is rapid, occurring in just a few days to weeks.^1,29–30^ For example, in healthy individuals subjected to 12 weeks of bed rest, left ventricualr mass index decreased 15%,^31^ and 25% declines in left ventricualr mass have been documented in spinal cord injury patients.^32^ Clinically, cardiac mass decreases 40% to 50% after the obstruction of aortic stenosis is relieved, a process that is accompanied by improvement in heart function.^33–34^ Astronauts exhibit signs of cardiac deconditioning following prolonged exposure to zero gravity during spaceflight.^31^ Marked myocardial atrophy can be observed in patients treated with VAD therapy.^35^ Despite the importance of cardiac atrophy across multiple clinically relevant circumstances, relatively little is known about mechanisms governing the atrophic process.

The 2 major catabolic processes in cardiac muscle are the UPS and the autophagy‐lysosome pathway. Indeed, activation of these cellular cascades occurs in virtually all forms of cardiac plasticity; when the heart grows, anabolic pathways predominate, but when the heart shrinks, catabolic events are dominant. Importantly, some evidence, including data reported here, suggests that the relative importance of UPS and autophagic mechanisms are context dependent, raising the prospect that specifically targeting them in high‐risk patients may afford benefit.

Myocardial atrophy is also seen in the contexts of nutrient starvation or cancer cachexia.^36^ However, rather than being triggered by hemodynamic unloading, the atrophy response results from a variety of metabolic events, and the term “metabolic unloading” has been coined to describe this response.^37^ In contrast to mechanical unloading, the UPS appears to play a dominant role relative to autophagy in starvation‐induced cardiac atrophy. Interestingly, bariatric surgery in morbidly obese patients normalizes the overfed state and is associated with improvement in ventricular function^38^ and declines in cardiac mass.^39^ In other words, removal of excess nutrients, a state of relative starvation, appears to be beneficial to cardiac function in obese patients.

Whereas many studies have documented improvement in pathological cardiac hypertrophy in response to VAD therapy,^40–42^ underlying mechanisms are unclear. Ventricular mass and cardiomyocyte size decrease,^8^ contractile performance and left ventricular end‐diastolic dimensions trend toward normal,^42–43^ abnormalities in Ca^2+^ homeostasis improve,^44^ extracellular matrix remodels,^45^ and β‐adrenergic signaling is enhanced.^46^ In fact, in rare instances, the unloaded LV can improve sufficiently that explantation of a VAD is well tolerated.^47^ However, some effects of unloading are maladaptive. For example, mechanical unloading with a VAD can lead to disuse atrophy of a previously hypertrophied ventricle.^35^ For all these reasons, there is great interest in elucidating molecular events governing the remodeling of mechanically unloaded LV.

### Molecular Features of Cardiac Atrophy

Muscle atrophy is an active, energy‐requiring process whose mechanisms are just now being deciphered. In skeletal muscle, atrophy is controlled by pathways requiring activation of ubiquitin ligases (“atrogenes”).^48–49^ In fact, in the context of skeletal muscle atrophy, proteasome‐dependent catabolic pathways predominate.^50^ With respect to cardiac muscle, a number of negative regulators of growth—acting either through suppression of progrowth pathways or through direct stimulation of protein degradation—have been identified.^51^ However, the role of the UPS in cardiac atrophy is relatively less than that of autophagy, an assertion supported by findings reported here and the fact that MuRF1 levels increase only modestly in VAD‐treated hearts.^52^ These findings contrast with skeletal muscle atrophy, where expression of these ligases increases substantially in the setting of denervation‐induced unloading.^53–54^

Work reported here seeks to clarify the role of autophagy in cardiac atrophy. Autophagy has been studied in human hearts before and after VAD implantation. Unexpectedly, autophagy appears to be downregulated after VAD placement and mechanical unloading,^55^ although we have found evidence of FoxO3 activation. It is established that autophagic activity is elevated in the failing LV, possibly as an adaptive mechanism that promotes maintenance of cardiac function. VAD unloading rescues the failing heart, potentially obviating the need for high‐level autophagic flux. Evidence presented here reveals that autophagy is upregulated when a normal, healthy heart is unloaded.

### FoxO3‐Dependent Governance of Catabolism

Expressed at high levels in the heart, FoxO factors are characterized by a conserved 110–amino acid DNA‐binding motif called the “forkhead box” or “winged helix” domain.^56–57^ The FoxO family comprises 4 members (FoxO1, FoxO3, FoxO4, and FoxO6).^56,58^ Cardiomyocyte‐specific overexpression of FoxO1 is embryonic lethal,^59^ and skeletal myocyte–specific overexpression of FoxO3 triggers severe muscle atrophy.^60^ In *Caenorhabditis elegans*, the ortholog of FoxO, *daf‐16*, controls dauer formation, a state of developmental arrest in response to environmental cues such as starvation and overcrowding.^61^ This finding has led to the discovery of counterbalancing interactions between the signaling cascades governed by FoxO and insulin,^16,62–63^ which are activated by nutrient deprivation and surplus, respectively.

FoxO proteins activate the 2 major mechanisms of protein catabolism in muscle: UPS‐dependent degradation and autophagy.^10,64^ Under conditions of skeletal muscle unloading, for example, FoxO activates flux through both UPS and autophagic cascades, and the combination triggers muscle shrinkage.^10,64^ Some evidence in cultured neonatal cardiomyocytes suggests that FoxO1 and FoxO3 are capable of triggering autophagy, as well.^17^ In a recent report describing reversible overexpression of caFoxO3 in heart, early mortality was not observed.^65^ At this time, an obvious explanation for this discrepancy with our findings is not apparent, although technical and/or strain differences may contribute. In any event, before these 2 studies, nothing was known regarding the potential involvement of FoxO transcription factors in cardiac growth and atrophy in vivo or its potential involvement in mechanical unloading of the LV.

The predominant mechanism of FoxO transcription factor regulation is by posttranslational modification of FoxO proteins.^6^ In other words, the transcriptional activity of these proteins is regulated by a number of tractable signaling pathways. Given this, FoxO factors are a target of potential therapeutic interest in the control of cardiac mass, morphology, and function.

### Bnip3 as a FoxO3‐Dependent Activator of Autophagy

Autophagy is an evolutionarily conserved catabolic process whereby cells respond to energy stress by recycling intracellular components.^66^ In the setting of cardiac stress, activation of autophagic flux pathways occurs across a spectrum.^67^ At one end, low‐level constitutive autophagic flux is required for cell survival. At the other end of the spectrum, overactive autophagy can deplete a cell of elements required for life, thereby triggering cell death. In between these extremes, the actions of autophagic flux are complex and potentially prosurvival or antisurvival. The regulation of autophagy in the heart, however, is largely unknown. That said, recent scientific advances have raised the tantalizing prospect of targeting the myocyte autophagic reaction as a novel means of achieving therapeutic gain.^68^

Bnip3 is a BH3‐only Bcl2‐family protein known to be a downstream target of FoxO3.^64^ A major action of the BH3‐only proteins is proapoptotic, as they interact with and activate family members of the proapoptotic Bcl2 subfamily, such as Bax, Bak, and Bok. These molecules form oligomers, which are inserted into the mitochondrial outer membrane, leading to the release of apoptotic factors such as cytochrome C (see review^69^). However, appreciation of substantial functional variability among these molecules is starting to emerge.

Muscle is the body's largest reservoir of amino acids. From a teleological standpoint, it is possible to rationalize that BH3‐only proteins such as Bnip3 promote autophagy rather than apoptosis in muscle cells, as recycling of nutrients in muscle is needed under stress conditions. Numerous studies have identified Bnip3 as an effector of autophagy.^9,25,70^ Some studies have shown that Bnip3 competes with Beclin 1 for binding to Bcl2, relieving Beclin 1 of the inhibitory effects of Bcl2 binding and thereby promoting autophagy.^71^ In the present study, *Beclin 1* heterozygosity did not reverse the cardiac atrophy induced by activation of caFoxO3, suggesting that a single *Beclin 1* allele is sufficient to mediate the augmented autophagy induced by caFoxO3. Alternatively, there may be other pathways that mediate the autophagy process. Generation of cardiomyocyte‐specific *Beclin 1* knockouts will be required to elucidate whether Bnip3 acts solely through activating *Beclin 1*. This report is the first to establish a FoxO3‐Bnip3‐autophagy axis in adult hearts in vivo.

Bnip3 has been demonstrated to function mainly as a factor induced in response to ischemia^72–73^; it is barely detectable at either protein or mRNA levels in normal myocardium, and germline deletion of Bnip3 has no deleterious effect.^26^ Our results support the notion that, as with other BH3‐only proteins, Bnip3 must be regulated precisely, as these proteins are positioned at the crossroads of cellular life or death.

Overexpression of Bnip3 in the heart shortly after birth triggers dilated cardiomyopathy starting at 10 weeks of age.^26^ This stands in contrast with the cardiac atrophy phenotype induced by caFoxO3 activated in young adult mice, even though Bnip3 levels were increased dramatically, as well as our observation that Bnip3‐null mice were resistant to the atrophy‐inducing effect of caFoxO3. These differences may arise from the established differences between activating and deactivating cardiac genes conditionally, as opposed to their being driven by α*MHC Cre* (ie, expression starting shortly after birth).^74^ Nevertheless, the underlying mechanisms remain unclear. Also, the timing of Bnip3 overexpression could explain the different cardiac phenotypes. Interestingly, overexpression of cardiac Bnip3 shortly after birth induced minimal increases in cardiac apoptosis.^26^ The status of autophagy in these mice, however, was not studied. Thus, it is possible that enhanced autophagy may have contributed to the gradual development of cardiac dysfunction.

Whether increased expression of Bnip3 in response to pathological stimuli, such as ischemia or pressure overload, is beneficial or detrimental remains to be established. Studies by Diwan et al^26^ demonstrated that knockdown of Bnip3 in the heart was associated with beneficial postinfarction ventricular remodeling and improved contractile performance but not infarction size, possibly owing to the blunting of a Bnip3‐dependent autophagic response triggered by ischemia/reperfusion.^75^

### FoxO3‐Dependent Activation of the UPS

FoxO3 is known to promote activation of the UPS; consistent with this, we detected evidence of late degradation of the cardiomyocyte sarcomere. No longer viewed as static structures, sarcomeres are highly dynamic, subject to ongoing, carefully orchestrated remodeling involving protein synthesis and degradation. In recent years, it has become apparent that the UPS is the major mechanism to degrade sarcomeric proteins in both skeletal and cardiac muscle.^28,53,76–77^ The degradation process is initiated by ubiquitination of the contractile proteins catalyzed by “atrogenes” such as *Atrogin1* and *MuRF1*, both of which are FoxO3 targets (see review^78^). Interestingly, cardiomyocyte overexpression of *MuRF1* or *Atrogin1* rendered hearts susceptible to pressure‐overload heart failure.^79–80^ However, cardiac mass was not significantly different from WT under resting conditions. Together, these data are consistent with our finding that the autophagy‐lysosomal pathway is the major contributor to cardiac atrophy in adult mice triggered by caFoxO3, even though both autophagic and proteasomal pathways were activated at the same time.

cTnI is a major target of MURF1. Interestingly, we detected similarities in the cardiac phenotypes and sarcomere abnormalities induced by caFoxO3 with those elicited by deletion of the cTnI gene^81^ For example, in both contexts, myocyte ultrastructure is marked by shortened sarcomere length. In our study, inhibition of the proteasome temporarily improved cardiac function, suggesting strongly that UPS‐mediated degradation of sarcomeric proteins is responsible for the development of heart failure and lethality. Also, there was synergistic development of increased total protein ubiquitination, decreased cTnI levels, and cardiac dysfunction 72 hours after the initiation of caFoxO3 expression, followed by death within 24 to 48 hours. Based on these observations, we believe the cardiac dysfunction and fatality induced by caFoxO3 derived largely from sarcomere instability and derangement.

### Summary and Perspective

Findings reported here uncover FoxO3 as a master regulator of protein catabolism in the heart, orchestrating an atrophy response via both autophagy‐lysosomal and proteasomal degradation pathways. Intriguingly, the autophagy‐lysosomal pathway accounted for the majority of the cardiac atrophy phenotype induced by caFoxO3, whereas sarcomere degradation by proteasomal activation contributed to the emergence of cardiac dysfunction. These findings point to the possible dissociation of adaptive and maladaptive responses in the unloaded LV. Given that controlled, transient activation of atrophy‐promoting pathways in the pathologically hypertrophied heart has been postulated as a means to trigger declines in cardiac mass and improve symptoms and prognosis, these findings may lead to therapeutic options with clinical relevance.

## References

[1] Hill JA Olson EN Cardiac plasticity. N Engl J Med. 2008; 358:1370-1380 18367740 10.1056/NEJMra072139

[2] Katz AM Cardiomyopathy of overload. A major determinant of prognosis in congestive heart failure. N Engl J Med. 1990; 322:100-110 2403651 10.1056/NEJM199001113220206

[3] Okin PM Devereux RB Jern S Kjeldsen SE Julius S Nieminen MS Snapinn S Harris KE Aurup P Edelman JM Wedel H Lindholm LH Dahlof B Regression of electrocardiographic left ventricular hypertrophy during antihypertensive treatment and the prediction of major cardiovascular events. JAMA. 2004; 292:2343-2349 15547161 10.1001/jama.292.19.2343

[4] Burgering BM Kops GJ Cell cycle and death control: long live Forkheads. Trends Biochem Sci. 2002; 27:352-360 12114024 10.1016/s0968-0004(02)02113-8

[5] Accili D Arden KC FoxOs at the crossroads of cellular metabolism, differentiation, and transformation. Cell. 2004; 117:421-426 15137936 10.1016/s0092-8674(04)00452-0

[6] Ferdous A Battiprolu PK Ni YG Rothermel BA Hill JA FoxO, autophagy, and cardiac remodeling. J Cardiovasc Transl Res. 2010; 3:355-364 20577843 10.1007/s12265-010-9200-z PMC2994100

[7] Niimi M The technique for heterotopic cardiac transplantation in mice: experience of 3000 operations by one surgeon. J Heart Lung Transplant. 2001; 20:1123-1128 11595568 10.1016/s1053-2498(01)00309-6

[8] Bruckner BA Stetson SJ Perez‐Verdia A Youker KA Radovancevic B Connelly JH Koerner MM Entman ME Frazier OH Noon GP Torre‐Amione G Regression of fibrosis and hypertrophy in failing myocardium following mechanical circulatory support. J Heart Lung Transplant. 2001; 20:457-464 11295584 10.1016/s1053-2498(00)00321-1

[9] Aviv Y Shaw J Gang H Kirshenbaum LA Regulation of autophagy in the heart: “you only live twice”. Antioxid Redox Signal. 2011; 14:2245-2250 20712404 10.1089/ars.2010.3479

[10] Zhao J Brault JJ Schild A Cao P Sandri M Schiaffino S Lecker SH Goldberg AL FoxO3 coordinately activates protein degradation by the autophagic/lysosomal and proteasomal pathways in atrophying muscle cells. Cell Metab. 2007; 6:472-483 18054316 10.1016/j.cmet.2007.11.004

[11] Brunet A Bonni A Zigmond MJ Lin MZ Juo P Hu LS Anderson MJ Arden KC Blenis J Greenberg ME Akt promotes cell survival by phosphorylating and inhibiting a Forkhead transcription factor. Cell. 1999; 96:857-868 10102273 10.1016/s0092-8674(00)80595-4

[12] Sohal DS Nghiem M Crackower MA Witt SA Kimball TR Tymitz KM Penninger JM Molkentin JD Temporally regulated and tissue‐specific gene manipulations in the adult and embryonic heart using a tamoxifen‐inducible Cre protein. Circ Res. 2001; 89:20-25 11440973 10.1161/hh1301.092687

[13] Trotman LC Alimonti A Scaglioni PP Koutcher JA Cordon‐Cardo C Pandolfi PP Identification of a tumour suppressor network opposing nuclear Akt function. Nature. 2006; 441:523-527 16680151 10.1038/nature04809 PMC1976603

[14] Pinkston‐Gosse J Kenyon C DAF‐16/FOXO targets genes that regulate tumor growth in Caenorhabditis elegans. Nat Genet. 2007; 39:1403-1409 17934462 10.1038/ng.2007.1

[15] Ni YG Berenji K Wang N Oh M Sachan N Dey A Cheng J Lu G Morris DJ Castrillon DH Gerard RD Rothermel BA Hill JA Foxo transcription factors blunt cardiac hypertrophy by inhibiting calcineurin signaling. Circulation. 2006; 114:1159-1168 16952979 10.1161/CIRCULATIONAHA.106.637124 PMC4118290

[16] Ni YG Wang N Cao DJ Sachan N Morris DJ Gerard RD Kuro OM Rothermel BA Hill JA FoxO transcription factors activate Akt and attenuate insulin signaling in heart by inhibiting protein phosphatases. Proc Natl Acad Sci USA. 2007; 104:20517-20522 18077353 10.1073/pnas.0610290104 PMC2154463

[17] Sengupta A Molkentin JD Yutzey KE FoxO transcription factors promote autophagy in cardiomyocytes. J Biol Chem. 2009; 284:28319-28331 19696026 10.1074/jbc.M109.024406 PMC2788882

[18] Klionsky DJ Abeliovich H Agostinis P Agrawal DK Aliev G Askew DS Baba M Baehrecke EH Bahr BA Ballabio A Bamber BA Bassham DC Bergamini E Bi X Biard‐Piechaczyk M Blum JS Bredesen DE Brodsky JL Brumell JH Brunk UT Bursch W Camougrand N Cebollero E Cecconi F Chen Y Chin LS Choi A Chu CT Chung J Clarke PG Clark RS Clarke SG Clave C Cleveland JL Codogno P Colombo MI Coto‐Montes A Cregg JM Cuervo AM Debnath J Demarchi F Dennis PB Dennis PA Deretic V Devenish RJ Di Sano F Dice JF Difiglia M Dinesh‐Kumar S Distelhorst CW Djavaheri‐Mergny M Dorsey FC Droge W Dron M Dunn WA Jr Duszenko M Eissa NT Elazar Z Esclatine A Eskelinen EL Fesus L Finley KD Fuentes JM Fueyo J Fujisaki K Galliot B Gao FB Gewirtz DA Gibson SB Gohla A Goldberg AL Gonzalez R Gonzalez‐Estevez C Gorski S Gottlieb RA Haussinger D He YW Heidenreich K Hill JA Hoyer‐Hansen M Hu X Huang WP Iwasaki A Jaattela M Jackson WT Jiang X Jin S Johansen T Jung JU Kadowaki M Kang C Kelekar A Kessel DH Kiel JA Kim HP Kimchi A Kinsella TJ Kiselyov K Kitamoto K Knecht E Komatsu M Kominami E Kondo S Kovacs AL Kroemer G Kuan CY Kumar R Kundu M Landry J Laporte M Le W Lei HY Lenardo MJ Levine B Lieberman A Lim KL Lin FC Liou W Liu LF Lopez‐Berestein G Lopez‐Otin C Lu B Macleod KF Malorni W Martinet W Matsuoka K Mautner J Meijer AJ Melendez A Michels P Miotto G Mistiaen WP Mizushima N Mograbi B Monastyrska I Moore MN Moreira PI Moriyasu Y Motyl T Munz C Murphy LO Naqvi NI Neufeld TP Nishino I Nixon RA Noda T Nurnberg B Ogawa M Oleinick NL Olsen LJ Ozpolat B Paglin S Palmer GE Papassideri I Parkes M Perlmutter DH Perry G Piacentini M Pinkas‐Kramarski R Prescott M Proikas‐Cezanne T Raben N Rami A Reggiori F Rohrer B Rubinsztein DC Ryan KM Sadoshima J Sakagami H Sakai Y Sandri M Sasakawa C Sass M Schneider C Seglen PO Seleverstov O Settleman J Shacka JJ Shapiro IM Sibirny A Silva‐Zacarin EC Simon HU Simone C Simonsen A Smith MA Spanel‐Borowski K Srinivas V Steeves M Stenmark H Stromhaug PE Subauste CS Sugimoto S Sulzer D Suzuki T Swanson MS Tabas I Takeshita F Talbot NJ Talloczy Z Tanaka K Tanida I Taylor GS Taylor JP Terman A Tettamanti G Thompson CB Thumm M Tolkovsky AM Tooze SA Truant R Tumanovska LV Uchiyama Y Ueno T Uzcategui NL van der Klei I Vaquero EC Vellai T Vogel MW Wang HG Webster P Wiley JW Xi Z Xiao G Yahalom J Yang JM Yap G Yin XM Yoshimori T Yu L Yue Z Yuzaki M Zabirnyk O Zheng X Zhu X Deter RL Guidelines for the use and interpretation of assays for monitoring autophagy in higher eukaryotes. Autophagy. 2008; 4:151-175 18188003 10.4161/auto.5338 PMC2654259

[19] Zhu H Tannous P Johnstone JL Kong Y Shelton JM Richardson JA Le V Levine B Rothermel BA Hill JA Cardiac autophagy is a maladaptive response to hemodynamic stress. J Clin Invest. 2007; 117:1782-1793 17607355 10.1172/JCI27523 PMC1890995

[20] Okamoto K Kondo‐Okamoto N Ohsumi Y Mitochondria‐anchored receptor Atg32 mediates degradation of mitochondria via selective autophagy. Dev Cell. 2009; 17:87-97 19619494 10.1016/j.devcel.2009.06.013

[21] Calnan DR Brunet A The FoxO code. Oncogene. 2008; 27:2276-2288 18391970 10.1038/onc.2008.21

[22] Yang Z Klionsky DJ Mammalian autophagy: core molecular machinery and signaling regulation. Curr Opin Cell Biol. 2010; 22:124-131 20034776 10.1016/j.ceb.2009.11.014 PMC2854249

[23] Zhou J Liao W Yang J Ma K Li X Wang Y Wang D Wang L Zhang Y Yin Y Zhao Y Zhu WG FOXO3 induces FOXO1‐dependent autophagy by activating the AKT1 signaling pathway. Autophagy. 2012; 8:1712-1723 22931788 10.4161/auto.21830 PMC3541283

[24] Dorn GW II Kirshenbaum LA Cardiac reanimation: targeting cardiomyocyte death by BNIP3 and NIX/BNIP3L. Oncogene. 2008; 27suppl 1:S158-S167 19641501 10.1038/onc.2009.53

[25] Gustafsson AB Bnip3 as a dual regulator of mitochondrial turnover and cell death in the myocardium. Pediatr Cardiol. 2011; 32:267-274 21210091 10.1007/s00246-010-9876-5 PMC3051075

[26] Diwan A Krenz M Syed FM Wansapura J Ren X Koesters AG Li H Kirshenbaum LA Hahn HS Robbins J Jones WK Dorn GW Inhibition of ischemic cardiomyocyte apoptosis through targeted ablation of Bnip3 restrains postinfarction remodeling in mice. J Clin Invest. 2007; 117:2825-2833 17909626 10.1172/JCI32490 PMC1994631

[27] Mearini G Schlossarek S Willis MS Carrier L The ubiquitin‐proteasome system in cardiac dysfunction. Biochim Biophys Acta. 2008; 1782:749-763 18634872 10.1016/j.bbadis.2008.06.009

[28] Kedar V McDonough H Arya R Li HH Rockman HA Patterson C Muscle‐specific RING finger 1 is a bona fide ubiquitin ligase that degrades cardiac troponin I. Proc Natl Acad Sci USA. 2004; 101:18135-18140 15601779 10.1073/pnas.0404341102 PMC539735

[29] Levine BD Zuckerman JH Pawelczyk JA Cardiac atrophy after bed‐rest deconditioning: a nonneural mechanism for orthostatic intolerance. Circulation. 1997; 96:517-525 9244220 10.1161/01.cir.96.2.517

[30] Razeghi P Sharma S Ying J Li YP Stepkowski S Reid MB Taegtmeyer H Atrophic remodeling of the heart in vivo simultaneously activates pathways of protein synthesis and degradation. Circulation. 2003; 108:2536-2541 14610007 10.1161/01.CIR.0000096481.45105.13

[31] Perhonen MA Franco F Lane LD Buckey JC Blomqvist CG Zerwekh JE Peshock RM Weatherall PT Levine BD Cardiac atrophy after bed rest and spaceflight. J Appl Physiol. 2001; 91:645-653 11457776 10.1152/jappl.2001.91.2.645

[32] de Groot PC van Dijk A Dijk E Hopman MT Preserved cardiac function after chronic spinal cord injury. Arch Phys Med Rehabil. 2006; 87:1195-1200 16935054 10.1016/j.apmr.2006.05.023

[33] Iyem H Sekuri C Tavli M Buket S Left ventricular hypertrophy and remodeling after aortic valve replacement. Asian Cardiovasc Thorac Ann. 2007; 15:459-462 18042767 10.1177/021849230701500602

[34] Taniguchi K Takahashi T Toda K Matsue H Shudo Y Shintani H Mitsuno M Sawa Y Left ventricular mass: impact on left ventricular contractile function and its reversibility in patients undergoing aortic valve replacement. Eur J Cardiothorac Surg. 2007; 32:588-595 17689973 10.1016/j.ejcts.2007.07.003

[35] Kinoshita M Takano H Taenaka Y Mori H Takaichi S Noda H Tatsumi E Yagura A Sekii H Akutsu T Cardiac disuse atrophy during LVAD pumping. ASAIO Trans. 1988; 34:208-212 3196510

[36] Hellerstein HK Santiago‐Stevenson D Atrophy of the heart; a correlative study of 85 proved cases. Circulation. 1950; 1:93-126 15401199 10.1161/01.cir.1.1.93

[37] Baskin KK Taegtmeyer H Taking pressure off the heart: the ins and outs of atrophic remodelling. Cardiovasc Res. 2011; 90:243-250 21354996 10.1093/cvr/cvr060 PMC3115281

[38] Leichman JG Wilson EB Scarborough T Aguilar D Miller CC III Yu S Algahim MF Reyes M Moody FG Taegtmeyer H Dramatic reversal of derangements in muscle metabolism and left ventricular function after bariatric surgery. Am J Med. 2008; 121:966-973 18954843 10.1016/j.amjmed.2008.06.033 PMC2604808

[39] Algahim MF Lux TR Leichman JG Boyer AF Miller CC III Laing ST Wilson EB Scarborough T Yu S Snyder B Wolin‐Riklin C Kyle UG Taegtmeyer H Progressive regression of left ventricular hypertrophy two years after bariatric surgery. Am J Med. 2010; 123:549-555 20569762 10.1016/j.amjmed.2009.11.020 PMC2935191

[40] Margulies KB Reversal mechanisms of left ventricular remodeling: lessons from left ventricular assist device experiments. J Cardiac Fail. 2002; 8:S500-S505 10.1054/jcaf.2002.129264 12555165

[41] Burkhoff D Klotz S Mancini DM LVAD‐induced reverse remodeling: basic and clinical implications for myocardial recovery. J Card Fail. 2006; 12:227-239 16624689 10.1016/j.cardfail.2005.10.012

[42] Maybaum S Kamalakannan G Murthy S Cardiac recovery during mechanical assist device support. Semin Thorac Cardiovasc Surg. 2008; 20:234-246 19038734 10.1053/j.semtcvs.2008.08.003

[43] Maybaum S Mancini D Xydas S Starling RC Aaronson K Pagani FD Miller LW Margulies K McRee S Frazier OH Torre‐Amione G LVAD Working Group Cardiac improvement during mechanical circulatory support: a prospective multicenter study of the LVAD Working Group. Circulation. 2007; 115:2497-2505 17485581 10.1161/CIRCULATIONAHA.106.633180

[44] Chaudhary KW Rossman EI Piacentino V III Kenessey A Weber C Gaughan JP Ojamaa K Klein I Bers DM Houser SR Margulies KB Altered myocardial ca2+ cycling after left ventricular assist device support in the failing human heart. J Am Coll Cardiol. 2004; 44:837-845 15312868 10.1016/j.jacc.2004.05.049

[45] Bruckner BA Razeghi P Stetson S Thompson L Lafuente J Entman M Loebe M Noon G Taegtmeyer H Frazier OH Youker K Degree of cardiac fibrosis and hypertrophy at time of implantation predicts myocardial improvement during left ventricular assist device support. J Heart Lung Transplant. 2004; 23:36-42 14734125 10.1016/s1053-2498(03)00103-7

[46] Dipla K Mattiello JA Jeevanandam V Houser SR Margulies KB Myocyte recovery after mechanical circulatory support in humans with end‐stage heart failure. Circulation. 1998; 97:2316-2322 9639375 10.1161/01.cir.97.23.2316

[47] Dandel M Weng Y Siniawski H Stepanenko A Krabatsch T Potapov E Lehmkuhl HB Knosalla C Hetzer R Heart failure reversal by ventricular unloading in patients with chronic cardiomyopathy: criteria for weaning from ventricular assist devices. Eur Heart J. 2011; 32:1148-1160 20929978 10.1093/eurheartj/ehq353 PMC3086897

[48] Glass DJ Molecular mechanisms modulating muscle mass. Trends Mol Med. 2003; 9:344-350 12928036 10.1016/s1471-4914(03)00138-2

[49] Sandri M Sandri C Gilbert A Skurk C Calabria E Picard A Walsh K Schiaffino S Lecker SH Goldberg AL Foxo transcription factors induce the atrophy‐related ubiquitin ligase atrogin‐1 and cause skeletal muscle atrophy. Cell. 2004; 117:399-412 15109499 10.1016/s0092-8674(04)00400-3 PMC3619734

[50] Attaix D Ventadour S Codran A Bechet D Taillandier D Combaret L The ubiquitin‐proteasome system and skeletal muscle wasting. Essays Biochem. 2005; 41:173-186 16250905 10.1042/EB0410173

[51] Hardt SE Sadoshima J Negative regulators of cardiac hypertrophy. Cardiovasc Res. 2004; 63:500-509 15276475 10.1016/j.cardiores.2004.03.015

[52] Willis MS Rojas M Li L Selzman CH Tang R‐H Stansfield WE Rodriguez JE Glass DJ Patterson C Muscle ring finger 1 mediates cardiac atrophy in vivo. Am J Physiol Heart Circ Physio. 2009; 296:H997-H1006 10.1152/ajpheart.00660.2008 PMC2670686 19168726

[53] Bodine SC Latres E Baumhueter S Lai VK Nunez L Clarke BA Poueymirou WT Panaro FJ Na E Dharmarajan K Pan ZQ Valenzuela DM DeChiara TM Stitt TN Yancopoulos GD Glass DJ Identification of ubiquitin ligases required for skeletal muscle atrophy. Science. 2001; 294:1704-1708 11679633 10.1126/science.1065874

[54] Gomes MD Lecker SH Jagoe RT Navon A Goldberg AL Atrogin‐1, a muscle‐specific F‐box protein highly expressed during muscle atrophy. Proc Natl Acad Sci USA. 2001; 98:14440-14445 11717410 10.1073/pnas.251541198 PMC64700

[55] Kassiotis C Ballal K Wellnitz K Vela D Gong M Salazar R Frazier OH Taegtmeyer H Markers of autophagy are downregulated in failing human heart after mechanical unloading. Circulation. 2009; 120:S191-S197 19752367 10.1161/CIRCULATIONAHA.108.842252 PMC2778323

[56] Kaestner KH Knochel W Martinez DE Unified nomenclature for the winged helix/forkhead transcription factors. Genes Dev. 2000; 14:142-146 10702024

[57] Carlsson P Mahlapuu M Forkhead transcription factors: key players in development and metabolism. Dev Biol. 2002; 250:1-23 12297093 10.1006/dbio.2002.0780

[58] Wijchers PJ Burbach JP Smidt MP In control of biology: of mice, men and foxes. Biochem J. 2006; 397:233-246 16792526 10.1042/BJ20060387 PMC1513289

[59] Evans‐Anderson HJ Alfieri CM Yutzey KE Regulation of cardiomyocyte proliferation and myocardial growth during development by FOXO transcription factors. Circ Res. 2008; 102:686-694 18218983 10.1161/CIRCRESAHA.107.163428

[60] Kamei Y Miura S Suzuki M Kai Y Mizukami J Taniguchi T Mochida K Hata T Matsuda J Aburatani H Nishino I Ezaki O Skeletal muscle FOXO1 (FKHR) transgenic mice have less skeletal muscle mass, down‐regulated Type I (slow twitch/red muscle) fiber genes, and impaired glycemic control. J Biol Chem. 2004; 279:41114-41123 15272020 10.1074/jbc.M400674200

[61] Kenyon C Chang J Gensch E Rudner A Tabtiang R A C. elegans mutant that lives twice as long as wild type. Nature. 1993; 366:461-464 8247153 10.1038/366461a0

[62] Ogg S Paradis S Gottlieb S Patterson GI Lee L Tissenbaum HA Ruvkun G The Fork head transcription factor DAF‐16 transduces insulin‐like metabolic and longevity signals in C. elegans. Nature. 1997; 389:994-999 9353126 10.1038/40194

[63] Battiprolu PK Hojayev B Jiang N Wang ZV Luo X Iglewski M Shelton JM Gerard RD Rothermel BA Gillette TG Lavandero S Hill JA Metabolic stress‐induced activation of FoxO1 triggers diabetic cardiomyopathy in mice. J Clin Invest. 2012; 122:1109-1118 22326951 10.1172/JCI60329 PMC3287230

[64] Mammucari C Milan G Romanello V Masiero E Rudolf R Del Piccolo P Burden SJ Di Lisi R Sandri C Zhao J Goldberg AL Schiaffino S Sandri M FoxO3 controls autophagy in skeletal muscle in vivo. Cell Metab. 2007; 6:458-471 18054315 10.1016/j.cmet.2007.11.001

[65] Schips TG Wietelmann A Hohn K Schimanski S Walther P Braun T Wirth T Maier HJ FoxO3 induces reversible cardiac atrophy and autophagy in a transgenic mouse model. Cardiovasc Res. 2011; 91:587-597 21628326 10.1093/cvr/cvr144

[66] Mizushima N Levine B Cuervo AM Klionsky DJ Autophagy fights disease through cellular self‐digestion. Nature. 2008; 451:1069-1075 18305538 10.1038/nature06639 PMC2670399

[67] Rothermel BA Hill JA Autophagy in load‐induced heart disease. Circ Res. 2008; 103:1363-1369 19059838 10.1161/CIRCRESAHA.108.186551 PMC2607044

[68] Nemchenko A Chiong M Turer A Lavandero S Hill JA Autophagy as a therapeutic target in cardiovascular disease. J Mol Cell Cardiol. 2011; 51:584-593 21723289 10.1016/j.yjmcc.2011.06.010 PMC3177001

[69] Lomonosova E Chinnadurai G BH3‐only proteins in apoptosis and beyond: an overview. Oncogene. 2008; 27suppl 1:S2-S19 19641503 10.1038/onc.2009.39 PMC2928556

[70] Dorn GW II Mitochondrial pruning by Nix and BNip3: an essential function for cardiac‐expressed death factors. J Cardiovasc Transl Res. 2010; 3:374-383 20559783 10.1007/s12265-010-9174-x PMC2900478

[71] Zhang H Bosch‐Marce M Shimoda LA Tan YS Baek JH Wesley JB Gonzalez FJ Semenza GL Mitochondrial autophagy is an HIF‐1‐dependent adaptive metabolic response to hypoxia. J Biol Chem. 2008; 283:10892-10903 18281291 10.1074/jbc.M800102200 PMC2447655

[72] Kubli DA Quinsay MN Huang C Lee Y Gustafsson AB Bnip3 functions as a mitochondrial sensor of oxidative stress during myocardial ischemia and reperfusion. Am J Physiol Heart Circ Physiol. 2008; 295:H2025-H2031 18790835 10.1152/ajpheart.00552.2008 PMC2614576

[73] Band M Joel A Hernandez A Avivi A Hypoxia‐induced BNIP3 expression and mitophagy: in vivo comparison of the rat and the hypoxia‐tolerant mole rat, Spalax ehrenbergi. FASEB J. 2009; 23:2327-2335 19255257 10.1096/fj.08-122978

[74] Nakai A Yamaguchi O Takeda T Higuchi Y Hikoso S Taniike M Omiya S Mizote I Matsumura Y Asahi M Nishida K Hori M Mizushima N Otsu K The role of autophagy in cardiomyocytes in the basal state and in response to hemodynamic stress. Nat Med. 2007; 13:619-624 17450150 10.1038/nm1574

[75] Hamacher‐Brady A Brady NR Logue SE Sayen MR Jinno M Kirshenbaum LA Gottlieb RA Gustafsson AB Response to myocardial ischemia/reperfusion injury involves Bnip3 and autophagy. Cell Death Differ. 2007; 14:146-157 16645637 10.1038/sj.cdd.4401936

[76] Willis MS Ike C Li L Wang DZ Glass DJ Patterson C Muscle ring finger 1, but not muscle ring finger 2, regulates cardiac hypertrophy in vivo. Circ Res. 2007; 100:456-459 17272810 10.1161/01.RES.0000259559.48597.32 PMC4112093

[77] Witt CC Witt SH Lerche S Labeit D Back W Labeit S Cooperative control of striated muscle mass and metabolism by MuRF1 and MuRF2. EMBO J. 2008; 27:350-360 18157088 10.1038/sj.emboj.7601952 PMC2168395

[78] Zolk O Schenke C Sarikas A The ubiquitin‐proteasome system: focus on the heart. Cardiovasc Res. 2006; 70:410-421 16497285 10.1016/j.cardiores.2005.12.021

[79] Willis MS Schisler JC Li L Rodriguez JE Hilliard EG Charles PC Patterson C Cardiac muscle ring finger‐1 increases susceptibility to heart failure in vivo. Circ Res. 2009; 105:80-88 19498199 10.1161/CIRCRESAHA.109.194928 PMC2737442

[80] Li HH Kedar V Zhang C McDonough H Arya R Wang DZ Patterson C Atrogin‐1/muscle atrophy F‐box inhibits calcineurin‐dependent cardiac hypertrophy by participating in an SCF ubiquitin ligase complex. J Clin Invest. 2004; 114:1058-1071 15489953 10.1172/JCI22220 PMC522252

[81] Huang X Pi Y Lee KJ Henkel AS Gregg RG Powers PA Walker JW Cardiac troponin I gene knockout: a mouse model of myocardial troponin I deficiency. Circ Res. 1999; 84:1-8 9915769 10.1161/01.res.84.1.1

